# Protest against the Doctors’ Tax

**Published:** 1898-01

**Authors:** 


### Protest Against the Doctors' Tax.

The members of the medical fraternity in Texas propose to go before the State legislature during its next session and wage a bitter war against a continuance of the levying of the State occupation tax upon them. Many of them who attended the meeting in the council chamber of the city hall today to discuss this matter told a *News* reporter that while they could in their own minds partially justify the action of the State in charging members of other professions and occupations with a tax of this nature, they could not justify such an imposition on a man whose calling was that of saving the life of his fellow-men or the alleviation of their sufferings. The gist of what was done at the meeting is given below, but as many of the participants discussed the subject in an informal manner in the corridors of the city hall, and some of these discussions were overheard by a *News* reporter, the statement made in the beginning that a bitter war would be waged against the continuance of the tax is by the sentiments thus heard expressed, fully justified.

The call for today's meeting was issued about two months ago. The number of physicians who responded today, while not exceeding fifty, all told, represented every section of the State; and besides this, these fifty with the proxies which they held, represented something like 600 physicians, it is claimed.

The meeting was called to order by Dr. J. B. Smoot, of Dallas, and an organization effected by the election of Dr. J. E. Gibson, of McKinney, as permanent chairman and Dr. E. W. Capps, of Fort Worth, as permanent secretary. Then a recess was taken until 2 p. m.

Dr. Smoot opened the afternoon session with a statement giving the object of the meeting, and was followed by Dr. V. P. Armstrong, of Dallas, on whose motion the name of each delegate present who represented a medical association was furnished the Secretary. In carrying this motion it was developed that the representatives of eleven medical associations, with a membership amounting in the aggregate to nearly 600, were present.

Dr. J. H. Smart moved the appointment of a committee of five to hear the ideas and plans of those present for the accomplishment of the object in view. This carried, and the following doctors were accordingly made committeemen:

Moore of Van Alstyne, Cooper of Fort Worth, Benbrook of Rockwall, Hicks of Ellis County, and Smoot of Dallas.

The Chairman was, on motion, added to this committee.

The committee report, which was adopted, was as follows:

*To the Officers and Members of the Convention of Physicians of Texas, in Meeting Assembled:*

GENTLEMEN:—Your committee appointed to report upon the advisability of using our best efforts to have the occupation tax as now levied upon all members of our profession in Texas repealed, beg leave to report to you as follows: That representing the sentiment of the profession in Texas, we hereby declare said tax to be unjust and unwarranted. That we hereby call upon every member of the medical profession in Texas to proceed to use his earnest endeavors to have said tax duty repealed, resorting to whatever methods in their judgment may be most expedient to attain success. That this report be printed in circular form and be mailed to every county and city medical society in the State. That the Chairman of this convention be instructed to appoint a committee to carry into effect the provisions of this report. Respectfully submitted,

J. B. SMOOT,
Chairman, Dallas.
J. E. GIBSON, M. D.,
Collin County Medical Society, McKinney.
S. D. MOORE, M. D.,
Grayson County Medical Society, Van Alstyne, Tex.
A. S. HICKS, M. D.,
Griggs Medical Association, Ellis County.
J. T. BENBROOK, M. D.,
Rockwall County Medical Society, Rockwall, Tex.
J. L. COOPER, M. D.,
Secretary, Fort Worth, Texas.

In accordance with this report a committee consisting of Drs. Leflord, Wigin and Wynne, of Cherokee County, was appointed to carry out the plans suggested, and this committee was authorized to draw on the various district and county medical associations for the funds necessary to the discharge of their duties.

The meeting then adjourned sine die.—*Dallas News.*

